# Pathology features and the results of treatment of two cases of posterior choroidal leiomyoma

**DOI:** 10.1186/s12886-020-01445-6

**Published:** 2020-05-24

**Authors:** Nan Zhou, Wenbin Wei, Xiaolin Xu

**Affiliations:** Beijing Tongren Eye Center, Beijing Key Laboratory of Intraocular Tumor Diagnosis and Treatment, Medical Artificial Intelligence Research and Verification Laboratory of the Ministry of Industry and Information Technology, Beijing Tongren Hospital, Capital Medical University, Beijing, China

**Keywords:** Posterior choroidal leiomyoma, Pathology features, Local resection, Microinvasive vitrectomy, Case report

## Abstract

**Background:**

Posterior choroidal leiomyoma is an extremely rare tumor, to our knowledge, less than 10 cases reported in the literature. The definite diagnosis can be confirmed by immunohistochemistry, and local resection is preferable to enucleation for the posterior choroidal leiomyoma.

**Case presentation:**

Two adult Asian women presented with progressive vision loss in their right eyes. Ophthalmic examination revealed an amelanotic dome-shaped choroidal mass located in the fundus with yellowish exudative retinal detachment. Clinical differential diagnosis of a nonpigmented choroidal neoplasm mainly includes amelanotic melanoma, atypical hemangioma, metastatic carcinoma, as well as the rare posterior choroidal leiomyoma. Considering the choroidal lesion was more likely to be a benign tumor, then we performed the treatment of local resection by pars plana vitrectomy and the histopathological examination confirmed the diagnosis of choroidal leiomyoma. The best corrected visual acuity of the patients was more than 20/100 on 6-month follow-up.

**Conclusions:**

From these two posterior choroidal neoplasm cases, we were able to demonstrate local resection by the 23 to 25-gauge mircoinvasive vitrectomy for excision of intraocular tumors is a feasible treatment for choroidal leiomyoma.

## Background

Posterior choroidal leiomyoma (CL) is a rare tumor, to our knowledge, less than 10 cases reported in the literature [1–7]. Clinically, the diagnosis of CL is challenging and the differential diagnosis includes amelanotic choroidal melanoma, atypical hemangioma, schwannoma, neurilemmoma, and other neuroderived neoplasms in several clinicopathologic aspects. A rare variant of intraocular leiomyoma has been described with both myogenic and neurogenic histologic features [8], referred as mesectodermal leiomyoma because of its presumed origin from the neural crest. As for its rarity and ability to camouflage as melanoma, enucleation was common management for intraocular leiomyoma in most of the previous reports. Herein we report 2 cases of CL in young woman and the results of local excision treatment.

## Case presentation

### Case 1

**Fig. 1:**
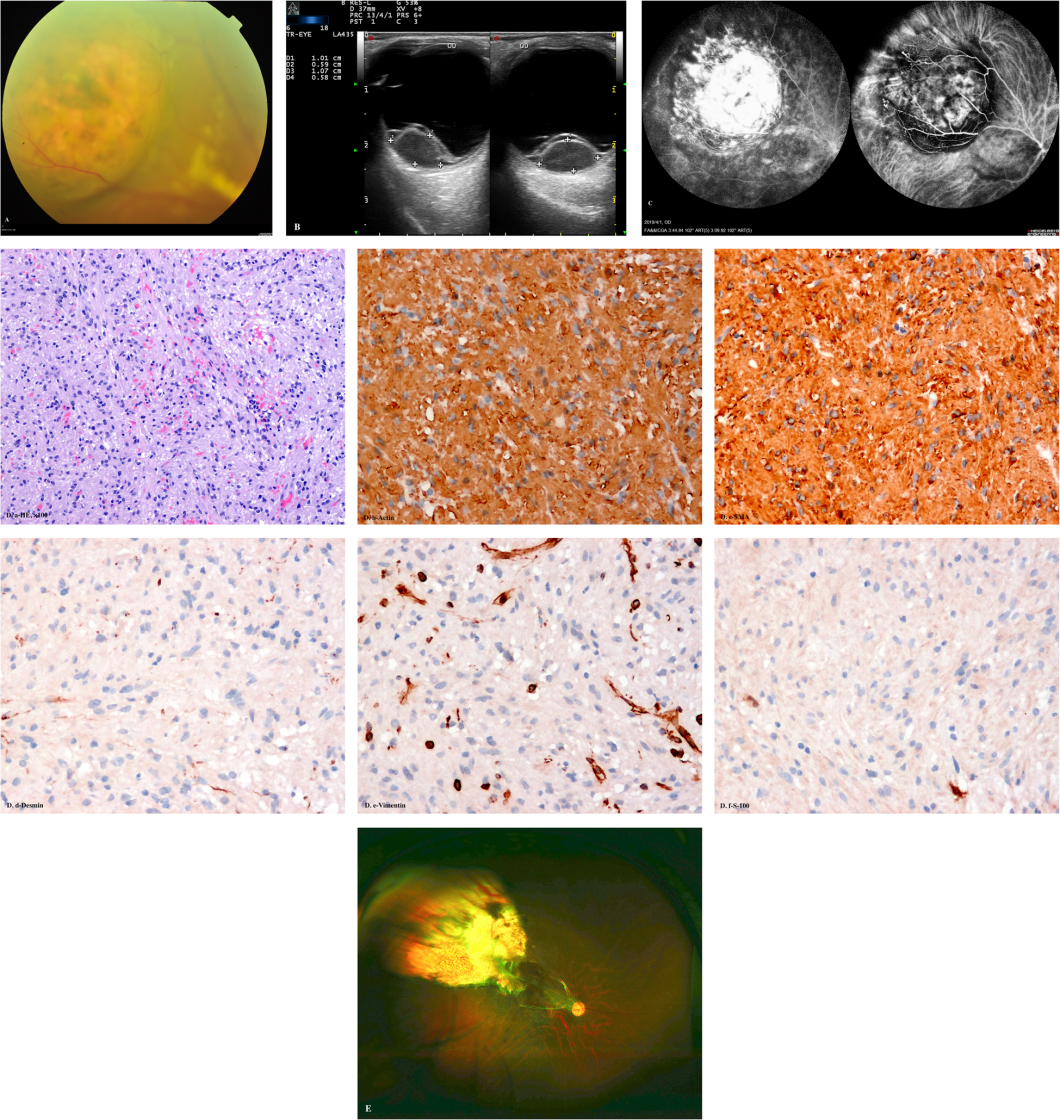
**a.** Fundus examination showed a large amelanotic dome-shaped non-pigmented choroidal tumor located the superotemporal quadrant with peripheral exudative retinal detachment involving the macula. **b.** Color doppler imaging (CDI) revealed pedunculated mass with inconsistent reflectivity of moderate intensity and no choroidal excavation, and arterial blood signals in the tumor. The size of the elevated lesion was 10.1× 5.9× 10.7 mm. **c.** On FFA the tumor demonstrated hypofluorescence in the early phase and hyperfluorescence with intense leakage on the surface in the late phase. On ICGA, the tumor showed hypofluorescence in the early phase and hyperfluorescence with prominent leakage in the late phase. **d.** Pathological features of posterior choroidal leiomyoma. a. Photomicrograph of leiomyoma showing loose cells with ovoid-shaped nuclei (HE, × 100). b-c. Tumor cells show positive immunoreactivity for Actin (b) and SMA (c) (peroxidase-antiperoxidase, × 200). d-f. Tumor cells show negative immunoreactivity for Desmin (d), Vimentin (e) and S-100 (f) (peroxidase-antiperoxidase, × 200). **e.** The retina was well attached with silicone oil in the last follow-up

A 34-year-old Asian woman presented with a 3-month history suffering of burred vison of the right eye. The woman had a history of small uterine fibroids and was not treated surgically. Dilated fundus examination showed a large amelanotic mushroom-shaped choroidal mass located the superotemporal quadrant with peripheral exudative retinal detachment involving the macula, and the tumor surrounded by yellowish intraretinal exudation (Fig. 1A). Color doppler imaging (CDI) demonstrated pedunculated mass with inconsistent reflectivity of moderate intensity and no choroidal excavation, and arterial blood signals in the tumor. The size of the elevated lesion was 10.1× 5.9× 10.7 mm (Fig. 1B). PET/CT scan was performed and excluded metastases. The clinical diagnoses included amelanotic choroidal melanoma, RPE adenoma, and hemangioma. Fluorescein fundus angiography (FFA) revealed the neoplasm hypofluorescence in the early stage and hyperfluorescence with intense leakage on the surface in the late stage. Indocyanine green angiography (ICGA) showed the neoplasm hypofluorescence in the early phase and hyperfluorescence with prominent leakage in the late phase (Fig. 1C). Considering the age of the patient, a benign diagnosis was favorable, and local resection by the 23-gauge mircoinvasive vitrectomy for excision of intraocular tumors was performed. As for the neoplasm was located in the posterior pole, it was difficult to perform transscleral transillumination, therefore, before the vitrectomy, we use endo-light source to conduct transillumination and find the neoplasm was transmitted light. The neoplasm was analyzed by pathology and immunohistochemistry. A well-defined pink choroidal tumor was found on the gross examination. The section of the tumor was white and soft. Under the light microscope (LM), the tumor is composed of spindle cells, which are arranged in fascicles, with a small amount of collagen deposition between cells. The nuclei were slightly pleomorphic and hyperchromatic, with occasional nucleoli and no atypical mitosis (Fig. 1D a). Immunohistochemical studies revealed that tumor cells show positive immunoreactivity for Actin and SMA (Fig. 1D b-c), negative immunoreactivity for Desmin (d), Vimentin (e), S-100 (Fig. 1D d-f) and HMB45/Melan-A. The diagnosis of CL was established, and the best corrected visual acuity was 20/66 after the vitrectomy. There was no found recurrence or metastases after 3 years follow-up.

### Case 2

**Fig. 2:**
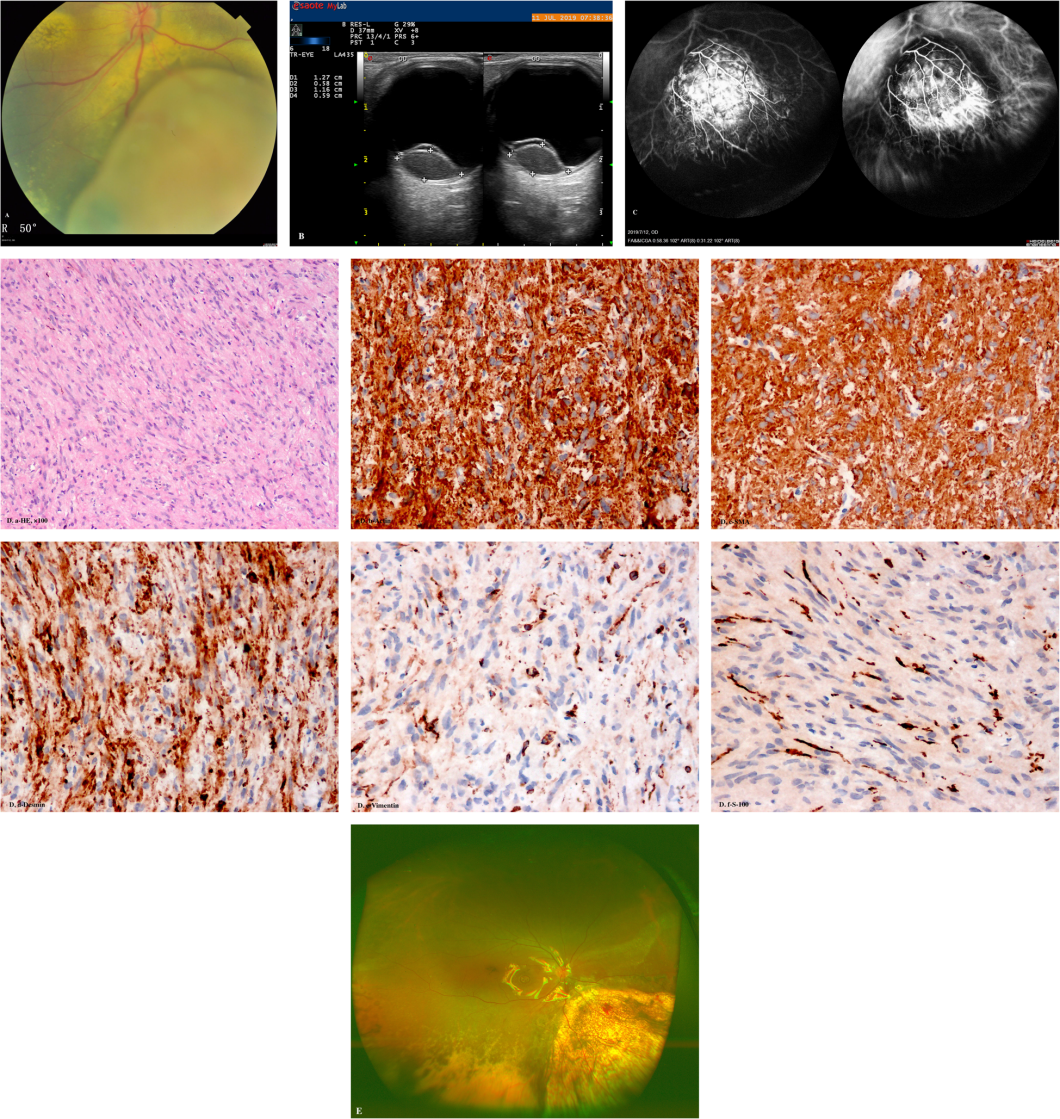
**a.** Clinical examination disclosed a yellowish-white choroidal mass located the inferior nasal quadrant of the right eye. Exudative retinal detachment and yellowish intraretinal exudation were also obverted. **b.** CDI was performed and showed a 12.7× 5.8× 11.6 mm tumoral mass without choroidal excavation. **c.** On FFA, the tumor showed hypofluorescence in the early phase and hyperfluorescence with strong leakage on the surface in the late phase, and the base of the tumor remained hypofluorescent. ICGA demonstrated hypofluorescence in the early phase and hyperfluorescence with obvious leakage in the late phase. Dual circulation could be seen. **d.** Pathological features of posterior choroidal leiomyoma. a. Photomicrograph of leiomyoma showing bundles of spindle cells with blunt-ended oval nuclei (HE, × 100). b-d. Tumor cells show positive immunoreactivity for Actin (b), SMA (c) and Desmin (d) (peroxidase-antiperoxidase, × 200). e-f. Tumor cells show negative immunoreactivity for Vimentin (e), and S-100 (f) (peroxidase-antiperoxidase, × 200). **e.** In the last follow-up, the retina was well attached with silicone oil and slight hemorrhage was observed in the defect area of retina and choroid

A 33-year-old Asian woman presented complaining of blurry vision for 2 weeks. The woman had no family history of systemic (uterine) fibroids or any subtle clinical signs associated with the disease. Fundus examination demonstrated a yellowish-white choroidal neoplasm located the inferior nasal quadrant of the right eye (Fig. 2A). Exudative retinal detachment and yellowish intraretinal exudation were also obverted. CDI was performed and showed a 12.7× 5.8× 11.6 mm tumoral mass without choroidal excavation (Fig. 2B). On FFA, the tumor showed hypofluorescence in the early stage and hyperfluorescence with strong leakage on the surface in the late stage. Moreover, the bottom of the tumor is still hypofluorescent. ICGA revealed hypofluorescence in the early stage and hyperfluorescence with obvious leakage in the late stage. Dual circulation of tumor vessels can be seen (Fig. 2C). 18F-FDG PET/CT has been underwent and no positive uptake in the other body parts. The diagnosis was not consistent with uveal melanoma or metastasis and the patient processed local resection by the 25-gauge mircoinvasive vitrectomy. The tumor exhibited marked translucency on transillumination. The tumor was sent for histopathological analysis, and the result demonstrated the tumor with same characteristics as case 1. Light microscopy showed a well-defined tumor in the posterior choroid, did not penetrate the sclera. The tumor consists of spindle cells arranged in bundles without mitosis. The background is fibrous, similar to glial tissue, and the cell outline is fuzzy. There were obvious capillary distribution between cell bundles (Fig. 2D. a). Immunohistochemistry showed that smooth muscle specific actin, SMA and Desmin were positive (Fig. 2D. b-d), while Vimentin, S-100 protein and Melan-A/HMB45 were negative (Fig. 2D. e-f). The diagnosis was choroidal leiomyoma. There was no signs of proliferative vitreoretinopathy or tumor recurrence after the surgery for 6 months of follow-up, and the best corrected visual acuity was 20/100.

## Discussion and conclusions

Posterior choroidal leiomyoma is extremely rare and is very difficult to be differentiated clinically, especially the amelanotic melanoma. Even with the magnetic resonance imaging (MRI), A- and B-scan ultrasonography [9] and fluorescein angiogram, CL still difficult to distinguish from amelanotic choroidal melanoma, RPE adenoma or metastasis. Thus far, there are less than 10 cases reported the neoplasm was located exclusively in the posterior choroid [1–7].

Of these cases of CL described above, 6 underwent enucleation due to the malignant tumors were considered to be diagnosed clinically [2, 3, 6, 7]. In another case, because a single case report without considering the malignant tumor occurred in a young patient, the biopsy specimens was performed using pars plana vitrectomy, thereby providing the correct diagnosis [5]. Shields et al. described the main clinical and pathological features of leiomyoma of the ciliary body and the choroid, based on 7 cases and 17 literature reviews [4]. In our two cases, compared with the previous personal observation results, some unique features to our case are described.

Microscopically, the intraocular tumor is composed of interlacing bundles of spindle cells and blunt-ended oval nuclei, with moderate amount of fibrous cytoplasm. On trichrome staining, there was no significant difference between intercellular myoglial fibrils and neurofibrils of Schwann cell tumor. Mitosis is rarely present. Many CL lesions showed a muscular appearance under light microscopy.

In addition, immunohistochemistry showed that the expression of smooth muscle actin and muscle specific actin were consistent in the two tumors mentioned in this report. The immunohistochemical results of our cases are consistent with those of smooth muscle tumors.

Local excision was performed in two patients by the 23 to 25-gauge mircoinvasive vitrectomy and reconstruction of the eyeball. No serious complications such as hemorrhage occurred during the operation. The retinas were well attached with silicone oil 3 months after operation. Follow-up for 2 years, there was no sign of tumor recurrences or retinal detachment. The best corrected visual acuity of the two patients were 20/66 and 20/100 respectively.

Neither of our patients developed metastasis.

In summary, we described 2 cases of CL, a rare, benign neoplasm which arises exclusively in the posterior choroid. Clinically, CL mimics malignant (metastasis and choroidal melanoma) and benign lesions. Under light microscopy, it is similar to the neural tumors, so muscular immunohistochemical markers and morphology should be carefully evaluated before the confirmed diagnosis.

And, if a tumor is suspected benign or malignant is uncertain, especially if the tumor is mainly located in the choroid, local resection is a reasonable treatment to its management, in that it preserves eye structure and remains visual function. However, optimal treatment methods of choroidal leiomyoma remain to be determined. More cases are needed to analyze the characteristics for more precise differentiation from other tumors, prognosis and appropriate therapeutic options.

## Data Availability

The data of this case report are available from the corresponding author on reasonable request.

## Abbreviations

BCVA: Best corrected visual acuity
CL: Choroidal leiomyoma
CDI: Colour doppler imaging
FFA: Fluorescein fundus angiography
ICGA: Indocyanine green angiography
LM: Light microscope
